# Effect of endo-1,4-xylanase and a complex of xylanase and β-glucanase supplementation on growth performance, energy utilization, and meat quality in broiler chickens

**DOI:** 10.14202/vetworld.2025.3929-3941

**Published:** 2025-12-14

**Authors:** Suvapit Visripat, Phiangchai Chailaor, Siriporn Namted, Choawit Rakangthong, Chanwit Kaewtapee, Chaiyapoom Bunchasak

**Affiliations:** 1 Department of Animal Science, Faculty of Agriculture, Kasetsart University, Bangkok, 10900, Thailand; 2 Department of Agriculture, Faculty of Agriculture Technology, Valaya Alongkorn Rajabhat University under the Royal Patronage, Pathum Thani, 13180, Thailand

**Keywords:** broiler chickens, carcass quality, endo-1,4-xylanase, energy utilization, feed efficiency, β-glucanase

## Abstract

**Background and Aim:**

Non-starch polysaccharides (NSP) in cereal-based poultry diets can impair nutrient digestibility and increase intestinal viscosity, reducing growth performance. Supplementation with NSP-degrading enzymes such as endo-1,4-xylanase and β-glucanase can improve energy utilization and feed efficiency. This study evaluated the effects of xylanase alone or in combination with β-glucanase on growth performance, carcass yield, and meat quality of broiler chickens fed energy-reduced diets.

**Materials and Methods:**

A total of 320 male Ross 308 broilers were distributed into four treatments with eight replicates of ten birds: Positive control (PC), negative control (NC; −100 kcal dietary metabolizable energy/kg), NC + xylanase (12.45 Internation Units [IU]/kg), and NC + xylanase + β-glucanase (12.45 + 12.8 IU/kg). Birds were reared for 37 days under a three-phase feeding program (starter, 1–10 days; grower, 11–24 days; finisher, 25–37 days). Growth indices, feed conversion ratio (FCR), energy conversion ratio (ECR), and European production efficiency factor (EPEF) were recorded. At 38 days, carcass yield, organ weights, and breast meat quality (pH, color, drip loss, wooden-breast, and white-striping scores) were assessed.

**Results:**

During the grower phase, enzyme supplementation significantly improved FCR, ECR, and EPEF compared with NC (p < 0.05). The combined xylanase + β-glucanase treatment produced energy-efficiency and cost benefits comparable to PC. In the finisher phase, xylanase alone improved FCR and ECR (p < 0.05). No significant effects on carcass yield, internal organ proportions, or breast meat quality were observed (p > 0.05). The incidence of wooden-breast and white-striping myopathies remained minimal across all groups.

**Conclusion:**

Supplementation with xylanase and β-glucanase enhances nutrient utilization and feed efficiency in low-energy corn–soybean diets without compromising carcass or meat quality. A combination of xylanase + β-glucanase is most beneficial during the grower phase, while xylanase alone optimizes performance during the finisher phase, providing a cost-effective and sustainable feeding strategy for broiler production.

## INTRODUCTION

The composition of poultry feed is largely determined by its starch and non-starch polysaccharide (NSP) content [1]. NSPs, which include both soluble and insoluble fractions, are not completely degraded by the endogenous enzymes of poultry and, therefore, reach the lower intestine undigested. Insoluble NSPs, typically present in cereal by-products, have a limited influence on digesta viscosity [2]. In contrast, soluble NSP are highly fermentable and can markedly increase intestinal viscosity, leading to reduced nutrient absorption and impaired growth performance in broilers [3].

To counteract these effects, exogenous enzymes are commonly incorporated into commercial diets rich in NSPs to lower digesta viscosity and enhance nutrient utilization. Supplementation with enzymes such as xylanase, β-glucanase, and β-mannanase has been shown to improve both nutrient digestibility [4] and the composition of the cecal microbiome in broiler chickens [5]. For instance, Munyaka *et al*. [6] demonstrated that adding a combination of endo-1,4-xylanase (2,500 U/kg) and endo-1,4-β-glucanase (250 U/kg) to corn- or wheat-based diets improved growth performance, reduced digesta viscosity, and increased nutrient digestibility. Similarly, Gilani *et al*. [7] reported that supplementation with endo-1,4-xylanase (1,220 U/kg) and endo-1,4-β-glucanase (152 U/kg) in diets with reduced digestible amino acids (−3.4%) and lower metabolizable energy (ME) (−105 kcal/kg from dietary fat) enhanced productive performance in broiler chickens.

Energy required for maintenance and growth is expressed as net energy (NE). In broilers, the efficiency of converting ME to NE differs by nutrient source, approximately 68% for digestible carbohydrates and 86% for digestible fat [8]. Thus, carbohydrates are less efficiently utilized for energy than fats (by 18% points). Because NSP-degrading enzymes primarily act on carbohydrate fractions rather than fat, evaluating their effect on dietary energy reduction should focus on carbohydrate-derived energy sources [7, 9].

Excessive growth rates in broilers are known to increase the incidence of breast muscle myopathies, such as wooden-breast (WB), which are closely associated with oxidative stress [10]. Under evaporative coolers, water is evaporated from saturated pads as air flows through, producing cooler, more humid air (evaporative-cooling systems). WB occurrence has been linked to approximately 14% excess body weight (BW) at slaughter [11]. It is hypothesized that metabolic overload, resulting from chronic oversupply of carbohydrates and fats, leads to lipotoxicity, glucotoxicity, and disruption of insulin-independent glucose transport, thereby contributing to WB and related myopathies in commercial broilers [12].

Although numerous studies have evaluated the benefits of NSP-degrading enzymes, such as xylanase and β-glucanase, in poultry nutrition, most have focused on their use in wheat- or barley-based diets or under standard ME conditions. Data on their efficacy in corn–soybean meal-based diets with reduced ME levels, which more accurately reflect commercial feed cost-reduction strategies, remain limited and inconsistent. Furthermore, the interactive effects of xylanase and β-glucanase supplementation at specific growth phases (starter, grower, and finisher) have not been clearly defined, especially when dietary energy is reduced from both starch and fat sources. The existing literature primarily assesses improvements in feed conversion ratio (FCR) or nutrient digestibility, but less attention has been given to carcass composition, breast meat quality, and myopathy incidence (WB and white-striping [WS]) in birds raised under tropical conditions, such as those prevalent in Thailand. The lack of data on how enzyme supplementation influences energy utilization efficiency, economic feed cost, and meat quality integrity in low-energy diets represents a significant research gap. Addressing this knowledge gap is essential to optimizing enzyme use in commercial broiler production systems that aim to balance feed efficiency, carcass quality, and production sustainability.

The present study aimed to evaluate the effects of supplementing endo-1,4-xylanase alone and in combination with endo-1,4-β-glucanase on the growth performance, carcass yield, and breast meat quality of broiler chickens fed reduced-energy corn–soybean meal diets. Specifically, the study sought to:

1. Assess how xylanase and xylanase + β-glucanase supplementation influence FCR, energy conversion ratio (ECR), and European production efficiency factor (EPEF) during different growth phases.
2. Determine whether enzyme supplementation can offset performance losses associated with a 100 kcal/kg reduction in apparent metabolizable energy (AME).
3. Evaluate the effects on carcass composition, internal organ development, and breast meat quality parameters, including pH, color, drip loss, and the incidence of WB and WS myopathies.
4. Propose phase-specific enzyme supplementation strategies to optimize energy utilization and maintain meat quality in broilers raised under tropical production conditions.

By addressing these objectives, the study provides novel insights into phase-targeted enzyme applications in low-energy diets, contributing to the development of cost-effective, sustainable feeding programs that preserve both productivity and product quality in modern broiler production.

## MATERIALS AND METHODS

### Ethical approval

All experimental procedures involving animals were reviewed and approved by the Institutional Animal Care and Use Committee of Kasetsart University, Thailand. The study was conducted in strict accordance with the ethical principles for animal experimentation established by the Office of the National Research Council of Thailand. Approval was granted under Protocol ID: ACKU66-AGR-012 and License No. U1-03966-2559, dated August 18, 2023.

The study complied with the Animal Research: Reporting of *In Vivo* experiments 2.0 guidelines for the reporting of animal research and followed national and institutional standards for the care and use of laboratory animals, ensuring minimal distress and optimal welfare throughout the experimental period.

### Study period and location

The study was conducted from November 21, 2023, to December 28, 2023, at the Luang Suwan Vajokkasikij Chicken Farm, Department of Animal Science, Faculty of Agriculture, Kasetsart University, Bangkok, Thailand. All management and environmental control systems followed standard commercial practices for broiler rearing under tropical conditions.

### Tested enzymes

Two commercial NSP-degrading enzyme products were evaluated:

- Xylanase: Supplied at a rate equivalent to *12.45 IU/kg feed* (based on 1,500 Endo-Pentosanase Units [EPU]/kg feed ÷ 120.482).
- β-glucanase (cellulase): Supplied at *12.80 International Unit [IU]/kg feed* (based on 100 Cellulase Unit [CU]/kg feed ÷ 7.8125).

These enzymes were incorporated into experimental diets either singly or in combination, as described below.

### Experimental animals and management

A total of 320 male Ross 308 broiler chicks, aged 1 day, were randomly assigned to four treatment groups using a completely randomized design (CRD) (Table 1). Each treatment consisted of 8 replicates, with 10 chicks per replicate.

**Table 1:** Experimental groups.

Group	Treatment groups
1	Positive control (PC): Basal diet with standard AME.
2	Negative control (NC): Basal diet with 100 kcal/kg reduction in AME.
3	NC + Xylanase (NC + X): NC diet supplemented with endo-1,4-xylanase.
4	NC + Xylanase + β-Glucanase (NC + X + G): NC diet supplemented with both enzymes.

Birds were reared in a floor-released system within a closed housing facility equipped with an evaporative-cooling tunnel ventilation system comprising four 50-inch exhaust fans. Lighting was provided for 24 h/day during the 1^st^ week, then reduced to 20 h/day thereafter.

Temperature and relative humidity were recorded three times daily (07:00, 12:00, and 17:00) at the front, middle, and rear of the facility. All birds were vaccinated against Newcastle disease and infectious bronchitis at 1 and 10 days of age. Feed and water were available *ad libitum* throughout the experiment.

### Experimental diets

The birds were fed corn–soybean meal-based diets formulated according to the Ross 308 nutrient recommendations [14] under a three-phase feeding program:

- Starter phase: 1–10 days (mash form)
- Grower phase: 11–24 days (pellet form)
- Finisher phase: 25–37 days (pellet form)

The treatment groups were as follows:

1. Positive control (PC): Basal diet with standard AME.
2. Negative control (NC): Basal diet with 100 kcal/kg reduction in AME.
3. NC + Xylanase (NC + X): NC diet supplemented with endo-1,4-xylanase.
4. NC + Xylanase + β-Glucanase (NC + X + G): NC diet supplemented with both enzymes.

To ensure comparability, the starch-to-fat ratio was maintained constant across all treatments. Diet compositions and calculated nutrient values for each phase are presented in Tables 2–4.

**Table 2:** Feed compositions (1–10 days).

Ingredients	Positive control	Negative control	Negative control + xylanase	Negative control + xylanase + β-glucanase
Corn	40.80	36.69	36.68	36.68
Wheat	8.00	8.00	8.00	8.00
Barley	7.00	7.00	7.00	7.00
Rice bran oil	1.65	1.65	1.65	1.65
Soybean meal 48% crude protein	32.61	31.50	31.50	31.50
Full-fat soybean	4.98	4.00	4.00	4.00
Wheat bran	-	6.00	6.00	6.00
60% choline chloride	0.02	0.03	0.03	0.03
Monocalcium phosphate (22% Phosphorus)	1.89	1.91	1.91	1.91
Limestone	1.44	1.45	1.45	1.45
Salt	0.49	0.57	0.57	0.57
DL-Methionine 99%	0.40	0.42	0.42	0.42
L-Lysine hydrochloride 78%	0.27	0.31	0.31	0.31
L-Threonine 99%	0.16	0.18	0.18	0.18
Premix	0.24	0.24	0.24	0.24
Monensin	0.05	0.05	0.05	0.05
1, 4-xylanase	-	-	0.01	-
1, 4-β-glucanase	-	-	-	0.01
Total	100.00	100.00	100.00	100.00
Feed cost/kg the (baht)	14.15	13.87	13.90	13.91
Nutrients by calculation				
Dietary metabolizable energy (Kcal/Kg)	3000.00	2904.91	2904.91	2904.91
Crude protein (%)	23.00	23.00	23.00	23.00
Crude fat (%)	5.11	4.95	4.95	4.95
Crude fiber (%)	3.20	3.54	3.54	3.54
Neutral detergent fiber (%)	9.02	10.39	10.39	10.39
Acid detergent fiber (%)	3.89	4.32	4.31	4.31
Starch (g)	34.51	33.25	33.24	33.24
Starch/fat ratio	6.75	6.72	6.72	6.72
Dry matter (%)	88.69	88.81	88.81	88.81
Moisture (%)	11.31	11.19	11.19	11.19
Ash (%)	5.77	5.84	5.84	5.84
Calcium (%)	1.00	1.00	1.00	1.00
Total Phosphorus (%)	0.79	0.82	0.82	0.82
Digestible Phosphorus (%)	0.48	0.48	0.48	0.48
Sodium (%)	0.20	0.23	0.23	0.23
Total Lysine (%)	1.44	1.43	1.43	1.43
Standardized ileal digestibility lysine (%)	1.28	1.28	1.28	1.28
Total methionine (%)	0.73	0.74	0.74	0.74
Standardized ileal digestibility methionine (%)	0.69	0.70	0.70	0.70
Total methionine + Cystine (%)	1.10	1.10	1.10	1.10
Standardized ileal digestibility methionine + Cystine (%)	0.95	0.95	0.95	0.95
Total Threonine (%)	1.02	1.00	1.00	1.00
Standardized ileal digestibility Threonine (%)	0.86	0.86	0.86	0.86
Dietary electrolyte balance (mEq/kg)	270.37	268.99	268.99	268.99

**Table 3:** Feed compositions (11–24 days).

Ingredients	Positive control	Negative control	Negative control + xylanase	Negative control + xylanase + β-glucanase
Corn	37.60	34.35	34.34	34.34
Wheat	10.00	10.00	10.00	10.00
Barley	10.00	10.00	10.00	10.00
Rice bran oil	2.50	2.50	2.50	2.50
Soybean meal 48% crude protein	25.89	25.25	25.25	25.25
Full-fat soybean	9.68	7.50	7.50	7.50
Wheat bran		5.96	5.96	5.96
60% choline chloride	0.05	0.06	0.06	0.06
Monocalcium phosphate (22% Phosphorus)	1.67	1.69	1.69	1.69
Limestone	1.30	1.31	1.31	1.31
Salt	0.49	0.49	0.49	0.49
DL-Methionine 99%	0.20	0.22	0.22	0.22
L-Lysine hydrochloride 78%	0.21	0.25	0.25	0.25
L-Threonine 99%	0.12	0.13	0.13	0.13
Premix	0.24	0.24	0.24	0.24
Monensin	0.05	0.05	0.05	0.05
1, 4-xylanase	-	-	0.01	-
1, 4-β-glucanase	-	-	-	0.01
Total	100.00	100.00	100.00	100.00
Feed cost/kg the (baht)	13.94	13.59	13.62	13.63
Nutrients by calculation				
Dietary metabolizable energy (Kcal/Kg)	3100.00	3000.00	3000.00	3000.00
Crude protein (%)	21.50	21.50	21.50	21.50
Crude fat (%)	6.67	6.30	6.30	6.30
Crude fiber (%)	3.32	3.63	3.63	3.63
Neutral detergent fiber (%)	9.41	10.77	10.76	10.76
Acid detergent fiber (%)	3.98	4.37	4.37	4.37
Starch (g)	35.16	34.42	34.41	34.41
Starch/fat ratio	5.27	5.46	5.46	5.46
Dry matter (%)	88.83	88.93	88.93	88.93
Moisture (%)	11.17	11.07	11.07	11.07
Ash (%)	5.32	5.39	5.39	5.39
Calcium (%)	0.90	0.90	0.90	0.90
Total Phosphorus (%)	0.72	0.76	0.76	0.76
Digestible Phosphorus (%)	0.44	0.43	0.43	0.43
Sodium (%)	0.20	0.20	0.20	0.20
Total Lysine (%)	1.31	1.29	1.29	1.29
Standardized ileal digestibility lysine (%)	1.15	1.15	1.15	1.15
Total methionine (%)	0.51	0.52	0.52	0.52
Standardized ileal digestibility methionine (%)	0.48	0.49	0.49	0.49
Total methionine + Cystine (%)	0.87	0.87	0.87	0.87
Standardized ileal digestibility methionine + Cystine (%)	0.72	0.71	0.71	0.71
Total Threonine (%)	0.93	0.91	0.91	0.91
Standardized ileal digestibility Threonine (%)	0.77	0.77	0.77	0.77
Dietary electrolyte balance (mEq/kg)	256.26	253.63	253.63	253.63

**Table 4:** Feed compositions (25–37 days).

Ingredients	Positive control	Negative control	Negative control + xylanase	Negative control + xylanase + β-glucanase
Corn	41.06	37.77	37.76	37.76
Wheat	10.00	10.00	10.00	10.00
Barley	10.00	10.00	10.00	10.00
Rice bran oil	3.20	3.20	3.20	3.20
Soybean meal 48% crude protein	18.86	18.00	18.00	18.00
Full-fat soybean	12.96	11.00	11.00	11.00
Wheat bran	0	6.00	6.00	6.00
60% choline chloride	0.07	0.08	0.08	0.08
Monocalcium phosphate (22% Phosphorus)	1.52	1.54	1.54	1.54
Limestone	1.15	1.15	1.15	1.15
Salt	0.22	0.24	0.24	0.24
DL-Methionine 99%	0.18	0.19	0.19	0.19
L-Lysine hydrochloride 78%	0.19	0.23	0.23	0.23
L-Threonine 99%	0.10	0.11	0.11	0.11
Premix	0.24	0.24	0.24	0.24
Monensin	0.25	0.25	0.25	0.25
1, 4-xylanase	-	-	0.01	-
1, 4-β-glucanase	-	-	-	0.01
Total	100.00	100.00	100.00	100.00
Feed cost/kg the (baht)	13.78	13.43	13.46	13.47
Nutrients by calculation				
Dietary metabolizable energy (Kcal/Kg)	3200.00	3100.00	3100.00	3100.00
Crude protein (%)	19.50	19.50	19.50	19.50
Crude fat (%)	7.96	7.64	7.64	7.64
Crude fiber (%)	3.35	3.67	3.67	3.67
Neutral detergent fiber (%)	9.47	10.84	10.84	10.84
Acid detergent fiber (%)	3.91	4.31	4.31	4.31
Starch (g)	37.26	36.50	36.49	36.49
Starch/fat ratio	4.68	4.78	4.78	4.78
Dry matter (%)	88.80	88.91	88.91	88.91
Moisture (%)	11.20	11.09	11.09	11.09
Ash (%)	4.82	4.90	4.90	4.90
Calcium (%)	0.80	0.80	0.80	0.80
Total Phosphorus (%)	0.67	0.70	0.70	0.70
Digestible Phosphorus (%)	0.40	0.40	0.40	0.40
Sodium (%)	0.16	0.17	0.17	0.17
Total Lysine (%)	1.17	1.15	1.15	1.15
Standardized ileal digestibility lysine (%)	1.02	1.02	1.02	1.02
Total methionine (%)	0.47	0.47	0.47	0.47
Standardized ileal digestibility methionine (%)	0.44	0.45	0.45	0.45
Total methionine + Cystine (%)	0.80	0.80	0.80	0.80
Standardized ileal digestibility methionine + Cystine (%)	0.65	0.65	0.65	0.65
Total Threonine (%)	0.82	0.81	0.81	0.81
Standardized ileal digestibility Threonine (%)	0.68	0.68	0.68	0.68
Dietary electrolyte balance (mEq/kg)	257.12	254.81	254.81	254.81

### Measurements of growth performance

Performance parameters were recorded for each feeding phase (starter, grower, and finisher). The following variables were measured:

BW and BW gain (BWG)
Feed intake (FI), energy intake (EI), starch intake, and fat intake
FCR, ECR, and feed cost per gain (FCG)
Mortality rate and BW uniformity (calculated as the coefficient of variation)

Overall production efficiency was expressed as the EPEF. The ECR was calculated as dietary EI per unit of BWG.

### Carcass yield measurements

At 38 days of age, four replicates per treatment group (a total of 160 birds) with uniform BWs were selected for carcass evaluation. Birds were euthanized by CO_2_ asphyxiation, followed by exsanguination. After plucking, the internal organs and abdominal fat were removed, and the carcass weight was recorded. Each carcass was divided into standard parts – outer breast, inner breast, wing, thigh, drumstick, and abdominal fat – and expressed as a percentage of live BW.

### Meat quality assessment

At 24 h postmortem, the right breast fillets (pectoralis major) were analyzed for pH and color (L*, a*, b*) using a Minolta colorimeter (Konica Minolta Business Solutions Co., Ltd., Thailand) at three dorsal points, while left fillets were used for dimensional measurements. The muscle pH was measured with a Testo spear-tip probe (Testo SE & Co. KGaA, Germany). Drip loss and color parameters were used as indicators of meat quality and freshness.

### Evaluation of WB and WS

Breast fillets were visually and manually assessed for WB and WS myopathies according to the classification scale of Oliveira *et al*. [15].

- Normal: Flexible throughout
- Moderate: Firmness in the mid-to-caudal region
- Severe: Hardness extending from cranial to caudal regions

**Figure 1:**
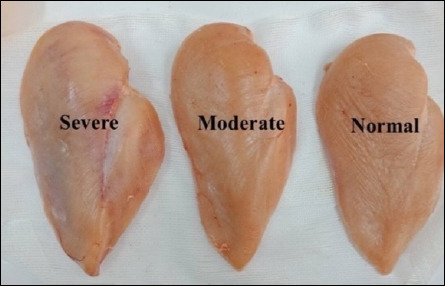
Wooden-breast scoring: Normal, moderate, and severe.

The mean values and standard deviations (SDs) for L*, a*, and b* parameters were calculated for each severity level (Table 5 and Figure 1). The incidence of WB and WS was reported as percentages of total samples evaluated [11, 16].

**Table 5:** Wooden-breast scoring.

Score	Category	Description
0	Normal breast meat	Flexible throughout the meat
1	Moderately wooden-breast	Flexible in the mid- to caudal regions
2	Severe wooden-breast	extremely hard from the cranial region to the caudal tip

### Statistical analysis

All data were analyzed using one-way analysis of variance, following a CRD with four treatments and eight replications per treatment. Results are expressed as mean ± SD. Significant differences among means (p < 0.05) were determined using Tukey’s multiple range test. Statistical analyses were performed using SAS University Edition software version 9.4 [17].

## RESULTS

### Growth performance

#### Starter phase (1–10 days)

The effects of supplementing endo-1,4-xylanase and a combination of xylanase and β-glucanase on broiler growth performance during the starter period are presented in Table 6. Supplementation with NSP-degrading enzymes resulted in lower BW (p < 0.05) compared with the PC group, although the NC group also exhibited a slightly reduced BW relative to the PC group. Xylanase supplementation tended to decrease FI (p = 0.09), whereas no significant effects of enzyme addition were observed on FCR, ECR, or other performance indicators.

**Table 6:** Effect of endo-1, 4-xylanase, endo 1, 4-xylanase, and endo-1, 4-β-glucanase supplementing in diets on growth performance of broiler chickens for 1–10 days.

Item	Positive control	Negative control	Negative control + xylanase	Negative control + xylanase + β-glucanase	p-value	SEM
Initial body weight (g)	44.34 ± 0.18	44.44 ± 0.18	44.34 ± 0.40	44.31 ± 0.20	0.78	0.26
Body weight (g)	339.58 ± 12.31^a^	330.48 ± 11.56^ab^	317.39 ± 16.83^b^	325.07 ± 10.91^b^	0.02	13.11
Average daily gain (g/day)	29.52 ± 1.23^a^	28.60 ± 1.15^ab^	27.31 ± 1.66^b^	28.08 ± 1.10^b^	0.02	1.3
Feed intake (g/day)	35.49 ± 1.37	36.15 ± 2.24	33.95 ± 1.32	35.07 ± 1.70	0.09	1.7
Energy intake (kcal/day)	106.46 ± 4.10^a^	105.00 ± 6.50^a^	98.63 ± 3.83^b^	101.89 ± 4.93^ab^	0.02	4.95
Starch intake (g/day)	12.25 ± 0.47^a^	12.02 ± 0.74^a^	11.29 ± 0.44^b^	11.66 ± 0.57^ab^	0.01	0.57
Fat intake (g/day)	1.81 ± 0.07^a^	1.79 ± 0.11^a^	1.68 ± 0.06^b^	1.74 ± 0.08^ab^	0.02	0.08
Feed conversion ratio	1.20 ± 0.01	1.26 ± 0.02	1.24 ± 0.02	1.25 ± 0.01	0.24	0.02
Energy conversion ratio	3.60 ± 0.10	3.67 ± 0.24	3.62 ± 0.22	3.63 ± 0.11	0.89	0.18
Feed cost per gain	17.02 ± 0.51	17.53 ± 0.17	17.33 ± 1.07	17.38 ± 0.53	0.69	0.65
European production efficiency factor	245.83 ± 5.28^a^	224.11 ± 6.44^b^	220.21 ± 8.24^b^	222.01 ± 4.27^b^	0.02	6.23
Mortality (%)	0.00 ± 0.00	0.00 ± 0.00	0.00 ± 0.00	1.25 ± 3.54	0.45	1.77
BW uniformity (%)	90.77 ± 2.10	91.67 ± 3.34	90.38 ± 2.20	91.61 ± 3.26	0.74	2.79

^a,b,c^Means within the same row with different superscripts are different (p < 0.05), SEM = Standard error of mean.

Overall, enzyme supplementation during the starter phase did not enhance early growth performance, suggesting limited enzyme activity or substrate availability at this stage.

#### Grower phase (11–24 days)

The results of enzyme supplementation on the growth performance of broilers during the grower phase are summarized in Table 7. During this period, the BW of birds fed the NC diet tended to be lower than that of the PC group (p = 0.09). However, supplementation with either xylanase alone or xylanase combined with β-glucanase significantly improved FCR and ECR and reduced FCG compared with the NC group (p < 0.05).

**Table 7:** Effect of supplementation with endo-1, 4-xylanase, endo 1, 4-xylanase, and endo-1, 4-β-glucanase on the growth performance of broiler chickens for 11–24 days.

Item	Positive control	Negative control	Negative control + xylanase	Negative control + xylanase + β-glucanase	p-value	SEM
Initial body weight (g)	339.58 ± 12.31a	330.48 ± 11.56ab	317.39 ± 16.83b	325.07 ± 10.91b	0.02	12.31
Body weight (g)	1,492.08 ± 42.21	1,439.71 ± 43.91	1,443.63 ± 49.85	1,480.47 ± 56.35	0.09	42.21
Average daily gain (g/day)	82.32 ± 2.56	79.23 ± 2.79	80.45 ± 2.89	82.53 ± 3.77	0.12	2.56
Feed intake (g/day)	103.10 ± 2.15	103.01 ± 4.28	99.40 ± 5.12	100.52 ± 4.84	0.24	2.15
Energy intake (kcal/day)	319.62 ± 2.36a	309.04 ± 4.54ab	298.21 ± 5.44b	301.57 ± 5.13b	0.01	2.36
Starch intake (g/day)	36.25 ± 0.26a	35.46 ± 0.52ab	34.21 ± 0.62b	34.59 ± 0.59b	0.04	0.26
Fat intake (g/day)	6.88 ± 0.05a	6.49 ± 0.96b	6.26 ± 0.11b	6.33 ± 0.10b	0.01	0.05
Feed conversion ratio	1.25 ± 0.04ab	1.30 ± 0.07a	1.24 ± 0.05b	1.22 ± 0.07b	0.05	0.04
Energy conversion ratio	3.88 ± 0.13a	3.90 ± 0.20a	3.70 ± 0.13b	3.65 ± 0.19b	0.01	0.13
Feed cost per gain	17.47 ± 0.62ab	17.69 ± 0.95a	16.83 ± 0.63ab	16.63 ± 0.91b	0.04	0.62
European production efficiency factor	648.92 ± 11.04ab	603.52 ± 19.58b	651.89 ± 12.60a	661.16 ± 18.20a	0.07	11.04
Mortality (%)	0.00 ± 0.00	0.00 ± 0.00	0.00 ± 0.00	1.25 ± 3.54	0.41	0
Culling (%)	1.250 ± 3.54	1.250 ± 3.54	0.00 ± 0.00	1.250 ± 3.54	0.80	3.54

^a,b,c^Means within the same row with different superscripts are differ (p < 0.05), SEM = Standard error of mean.

Notably, the ECR of the NC + xylanase + β-glucanase group was significantly better than that of the PC group (p < 0.01). The EPEF was lowest in the NC group (p = 0.02), whereas supplementation with both enzymes restored EPEF values to levels comparable to those in the PC group. FI, mortality rate, and BW uniformity were not significantly influenced by dietary treatment.

These findings indicate that enzyme supplementation effectively enhanced energy utilization and feed efficiency during the grower phase.

#### Finisher phase (25–37 days)

Table 8 presents the results for the finisher phase. Supplementation with xylanase alone resulted in a significant improvement in FCR (p < 0.05) and a reduction in FCG (p < 0.01) compared with the PC group. Both the NC and NC + xylanase groups showed lower FCG values than the PC diet, while the NC + xylanase group demonstrated a slightly higher average daily gain (ADG), EPEF, and BW uniformity (p > 0.05).

**Table 8:** Effect of supplementation with endo-1, 4-xylanase, endo 1, 4-xylanase, and endo-1, 4-β-glucanase supplementing in diet on the growth performance of broiler chickens for 25–37 days.

Item	Positive control	Negative control	Negative control + xylanase	Negative control + xylanase + β-glucanase	p-value	SEM
Initial body weight (g)	1,492.08 ± 42.21	1,439.71 ± 43.91	1,443.63 ± 49.85	1,480.47 ± 56.35	0.09	42.69
Body weight (g)	2,935.52 ± 66.62	2,976.67 ± 134.84	3,023.97 ± 67.14	2,998.08 ± 112.57	0.36	44.17
Average daily gain (g/day)	103.10 ± 2.45b	109.78 ± 8.69ab	112.88 ± 4.11ab	108.40 ± 5.54a	0.01	1.76
Feed intake (g/day)	186.48 ± 13.72	188.02 ± 8.61	185.25 ± 6.75	188.07 ± 7.17	0.92	5.53
Energy intake (Kcal/day)	596.75 ± 15.52	582.88 ± 9.44	574.26 ± 7.40	583.02 ± 7.86	0.52	4.49
Starch intake (g/day)	69.49 ± 5.11	68.63 ± 3.14	67.60 ± 2.46	68.63 ± 2.61	0.76	1.52
Fat intake (g/day)	14.84 ± 1.09	14.36 ± 0.65	14.15 ± 0.51	14.37 ± 0.54	0.31	0.29
Feed conversion ratio	1.68 ± 0.12a	1.59 ± 0.06ab	1.53 ± 0.08b	1.62 ± 0.12ab	0.03	0.03
Energy conversion ratio	5.37 ± 0.37a	4.94 ± 0.19b	4.73 ± 0.23b	5.00 ± 0.37b	0.05	0.05
Feed cost per gain	23.15 ± 0.56a	21.41 ± 0.30b	20.59 ± 0.36b	21.84 ± 0.57ab	0.05	0.28
European production efficiency factor	663.69 ± 46.27b	744.50 ± 88.67a	799.52 ± 63.92a	728.19 ± 89.058ab	0.01	27.53
BW uniformity (%)	91.06 ± 2.32	92.52 ± 2.75	93.26 ± 1.82	93.52 ± 2.58	0.19	0.85
Initial BW (g)	1,492.08 ± 42.21	1,439.71 ± 43.91	1,443.63 ± 49.85	1,480.47 ± 56.35	0.09	42.69

SEM = Standard error of mean.

These results suggest that xylanase supplementation during the finisher phase optimizes nutrient utilization and feed efficiency, compensating for the 100 kcal/kg reduction in AME without negatively affecting growth consistency or survival.

### Carcass yield and internal organs

The effects of xylanase and β-glucanase supplementation on carcass characteristics and internal organ proportions at 37 days of age are shown in Table 9. No significant differences (p > 0.05) were observed in the relative percentages of outer breast, inner breast, thigh, drumstick, abdominal fat, gizzard, liver, pancreas, or cecum among treatment groups.

**Table 9:** Effect of endo-1, 4-xylanase and endo 1, 4-xylanase and endo-1, 4-β-Glucanase supplementing in diet on carcass and internal organs of broiler chickens for 1–37 days.

Item	Positive control	Negative control	Negative control + xylanase	Negative control + xylanase + β-glucanase	p-value	SEM
Outer breast meat (%)*	20.65 ± 1.31	20.58 ± 1.75	20.35 ± 1.33	20.91 ± 1.29	0.40	0.11
Inner breast area (%)	3.88 ± 0.47	3.77 ± 0.38	3.67 ± 0.60	3.76 ± 0.38	0.28	0.04
Thigh meat (%)*	11.58 ± 1.25	11.37 ± 0.92	11.42 ± 0.89	11.32 ± 0.81	0.68	0.08
Drumstick (%)*	9.06 ± 0.65	8.97 ± 0.64	8.84 ± 0.57	8.98 ± 0.56	0.43	0.05
Abdominal fat (%)	1.21 ± 0.34	1.25 ± 0.28	1.24 ± 0.30	1.36 ± 0.33	0.16	0.01
Gizzard (%)	0.81 ± 0.17	0.85 ± 0.18	0.89 ± 0.19	0.89 ± 0.21	0.17	0.02
Liver (%)	1.96 ± 0.24	2.02 ± 0.19	2.09 ± 0.35	2.07 ± 0.40	0.22	0.02
Pancreases (%)	0.17 ± 0.03	0.18 ± 0.03	0.18 ± 0.03	0.19 ± 0.03	0.20	0.002
Cecum (%)	0.48 ± 0.13	0.45 ± 0.13	0.53 ± 0.15	0.48 ± 0.15	0.12	0.01
Outer breast meat (%)*	20.65 ± 1.31	20.58 ± 1.75	20.35 ± 1.33	20.91 ± 1.29	0.40	0.11
Inner breast area (%)	3.88 ± 0.47	3.77 ± 0.38	3.67 ± 0.60	3.76 ± 0.38	0.28	0.04
Thigh meat (%)*	11.58 ± 1.25	11.37 ± 0.92	11.42 ± 0.89	11.32 ± 0.81	0.68	0.08
Drumstick (%)*	9.06 ± 0.65	8.97 ± 0.64	8.84 ± 0.57	8.98 ± 0.56	0.43	0.05

* without skin, SEM = Standard error of mean.

The inclusion of NSP-degrading enzymes in the reduced-energy diet (−100 kcal/kg AME) did not adversely affect carcass composition or internal organ development. Furthermore, enzyme supplementation reduced production costs without compromising yield parameters. These results align with previous findings by El-Sayed [26], who reported that xylanase supplementation to low-energy diets (−150 kcal/kg ME) had no effect on organ or carcass weights in broilers.

### Meat quality

The impact of dietary enzyme supplementation on breast meat quality and myopathy incidence is summarized in Table 10. There were no significant differences (p > 0.05) among treatments in *drip loss, pH, or meat color parameters (L, a*, and b). Similarly, the incidence and severity of WB and WS were unaffected by enzyme supplementation.

**Table 10:** Effect of endo-1, 4-xylanase and endo 1, 4-xylanase and endo-1, 4-β-Glucanase supplementing in diet on the meat quality of broiler chickens at 38 days.

Item	Positive control	Negative control	Negative control + xylanase	Negative control + xylanase + β-glucanase	p-value	SEM
Drip loss (%)	2.46 ± 0.30	2.80 ± 0.20	2.89 ± 0.25	2.92 ± 0.71	0.43	0.11
pH	7.14 ± 0.15	7.14 ± 0.07	7.11 ± 0.09	7.07 ± 0.17	0.86	0.03
L*	50.52 ± 1.37	51.01 ± 1.27	50.46 ± 1.45	50.62 ± 0.85	0.93	0.29
a*	1.60 ± 0.76	1.36 ± 0.52	1.83 ± 0.57	1.34 ± 0.60	0.65	0.14
b*	6.27 ± 0.16	6.82 ± 0.0.32	6.90 ± 0.87	6.26 ± 0.39	0.20	0.01
Wooden-breast (%)*	1.20 ± 0.36	1.02 ± 0.40	1.00 ± 0.42	1.17 ± 0.57	0.88	0.16
White strip (%)	1.51 ± 0.14	1.43 ± 0.26	1.28 ± 0.40	1.53 ± 0.17	0.54	0.09
Drip loss (%)	2.46 ± 0.30	2.80 ± 0.20	2.89 ± 0.25	2.92 ± 0.71	0.43	0.11
pH	7.14 ± 0.15	7.14 ± 0.07	7.11 ± 0.09	7.07 ± 0.17	0.86	0.03
L*	50.52 ± 1.37	51.01 ± 1.27	50.46 ± 1.45	50.62 ± 0.85	0.93	0.29
a*	1.60 ± 0.76	1.36 ± 0.52	1.83 ± 0.57	1.34 ± 0.60	0.65	0.14
b*	6.27 ± 0.16	6.82 ± 0.0.32	6.90 ± 0.87	6.26 ± 0.39	0.20	0.01
Wooden-breast (%)*	1.20 ± 0.36	1.02 ± 0.40	1.00 ± 0.42	1.17 ± 0.57	0.88	0.16

* without skin, SEM = Standard error of mean.

These findings indicate that reducing AME by 100 kcal/kg, even with the inclusion of NSP-degrading enzymes, did not compromise breast meat quality or increase myopathy occurrence, ensuring consistent meat appearance and physicochemical properties across all treatment groups.

## DISCUSSION

### Effect of energy reduction on early growth performance

Reducing AME by 100 kcal/kg in the NC diet led to a suppression of BW in chicks during the starter phase, even in groups supplemented with NSP-degrading enzymes. This finding aligns with the report of Gilani *et al*. [7], who observed that xylanase and β-glucanase supplementation in a low-energy diet reduction of 105 kcal AME/kg) did not significantly influence growth during the early stage. These results suggest that enzyme addition at this age cannot fully compensate for the energy deficit, likely because young birds have limited capacity to increase FI to meet their metabolic energy demands.

The lower growth response to enzyme supplementation may be attributed to two possible mechanisms:

1. Nutrient partitioning – Digestible nutrients liberated by enzyme action may have been utilized preferentially to support gut microbiota development rather than rapid body growth.
2. Energy expenditure for enzymatic synergy – The oligosaccharides released by endo-xylanase can stimulate β-glucanase activity, but this synergistic action itself requires additional metabolic energy [19].

These findings indicate that enzyme supplementation in early growth stages may not enhance performance unless the intestinal environment and enzyme-substrate interaction are sufficiently established.

### Improved energy utilization during the grower phase

During the grower phase (11–24 days), supplementation with exogenous enzymes markedly improved FCR, ECR, and EPEF, while reducing FCG compared with the NC diet. The combination of xylanase and β-glucanase provided superior energy conversion efficiency compared with both the PC and NC groups.

At this stage, diets contained a higher proportion of soluble NSP sources (25.96%), including wheat, wheat bran, and barley, than during the starter phase (16.65%), thus offering sufficient substrate for enzyme activity. The observed improvements in feed efficiency can be attributed to enhanced NSP hydrolysis and nutrient digestibility. Luo *et al*. [20] reported that xylanase supplementation at 1,000 IU/kg improved broiler performance, while Lamp *et al*. [21] found that β-glucanase supplementation in a low-energy diet (−150 kcal/kg ME) yielded growth responses similar to those of the PC.

The reduction in FI observed with enzyme supplementation is consistent with findings by Samarasinghe *et al*. [22], who noted that birds consume less feed when their nutrient requirements are efficiently met. Moreover, Chen *et al*. [18] showed that supplementing NSP enzymes effectively enhanced utilization of nutrients in the feedstuff. In ผthe present study, resulting broilers have sufficient nutrients in maintenance of normal growth as intake of less dietary energy, starch, and fat in the NC + X group when compared to the PC and NC groups. Thus, resulting tends to decrease in FCG as well when compared to other groups. In the present study, this efficient nutrient utilization resulted in an EPEF comparable to that of the PC group, demonstrating that enzymatic hydrolysis of NSPs compensates for moderate dietary energy deficits. These findings agree with previous reports of González-Ortiz *et al*. [23] and Alam *et al*. [24] indicating that enzyme supplementation enhances profitability and production efficiency in broilers. According to Huang *et al*. [25], who reported that the factors affecting enzyme responses to nutrient digestibility of broiler chickens are age, ingredients, size of gastrointestinal tract, production of endogenous enzymes, and feeding ingestion capacity.

### Phase-specific enzyme efficiency during the finisher period

In the finisher phase (25–37 days), supplementation with endo-1,4-xylanase alone significantly improved FCR and reduced FCG compared with the PC group, while the combination of xylanase and β-glucanase was most effective during the grower phase. This difference may be linked to substrate availability and enzyme interaction.

During 11–24 days of age, sufficient soluble NSPs were available for both enzymes to act synergistically, while at 25–37 days, the production of endogenous digestive enzymes (e.g., amylase, lipase) is more mature. Hence, xylanase alone was adequate to release additional energy from carbohydrate fractions without requiring β-glucanase supplementation.

The present study, therefore, suggests a phase-dependent strategy:

- Xylanase + β-glucanase for the grower phase to enhance energy utilization from soluble NSPs.
- Xylanase alone for the finisher phase to sustain feed efficiency and reduce feed cost.

This phase-specific supplementation maximizes energy recovery, supports optimal performance, and provides economic benefits for broiler producers.

### Mechanistic role of NSP-degrading enzymes

Xylanase and β-glucanase function by hydrolyzing soluble NSPs, such as arabinoxylans and β-glucans, that otherwise increase intestinal viscosity and impair nutrient absorption. Their enzymatic action reduces digesta viscosity, improves nutrient diffusion, and enhances the accessibility of digestive enzymes to encapsulated nutrients. In addition, the oligosaccharides released during NSP hydrolysis may serve as prebiotic substrates, promoting beneficial microbial populations and improving gut health.

Together, these effects enhance energy utilization efficiency and maintain growth performance in low-energy diets, particularly those rich in soluble NSPs such as wheat and barley.

### Carcass and internal organ traits

No significant differences in carcass yield or internal organ proportions were observed among treatments, corroborating findings by Singh *et al*. [27], who reported no changes in organ weights following xylanase supplementation. Similarly, Ohotuowo *et al*. [28] found that xylanase and glucanase supplementation, even without dietary energy reduction, improved dressing percentage and breast yield without adverse effects on abdominal fat.

These results indicate that NSP-degrading enzymes improve energy utilization and reduce feed cost without negatively impacting carcass characteristics, reinforcing their safety and efficacy in broiler nutrition.

### Effects on meat quality and myopathies

Reducing AME (from starch and fat) by 100 kcal/kg with enzyme supplementation did not influence breast meat pH, color, or drip loss, nor did it affect the incidence of WB or WS. Singh *et al*. [27] reported that xylanase supplementation in low-energy diets (reduction of 150 kcal/kg ME) increased drip loss, whereas Selim *et al*. [29] observed that reducing dietary fat energy increased drip loss by 36%–46%.

The contrasting findings highlight that the source of energy reduction (starch vs. fat) may influence meat quality outcomes. Zakaria *et al*. [30] suggested that reducing starch-derived energy rather than fat when using NSP-degrading enzymes preserves breast meat quality. Likewise, Allouche *et al*. [31] demonstrated that exogenous enzymes in low-energy corn–soybean diets did not alter pH, moisture, or protein content of broiler meat.

In this study, balanced energy reduction from both starch and fat, combined with enzyme supplementation, successfully maintained meat quality and minimized myopathy risk.

### Metabolic implications and physiological perspective

The maintenance of breast meat quality despite energy reduction supports the hypothesis that moderate energy restriction mitigates metabolic overload and oxidative stress, two factors implicated in WB and WS development. High-energy diets often promote lipotoxicity and glucotoxicity [10], disrupting muscle metabolism [12], and promoting oxidative damage [13].

The dual-source energy reduction applied here likely moderated these metabolic stresses while allowing enzyme-driven energy optimization. Improved nutrient digestibility during the grower phase did not translate to excessive growth during finishing, thus preventing the oxidative burden associated with rapid muscle development [13].

This study, therefore, proposes a sustainable feeding strategy integrating moderate dietary energy reduction with targeted enzyme supplementation to optimize feed efficiency, reduce oxidative stress, and safeguard meat quality in modern broiler production systems.

## CONCLUSION

The present study demonstrated that supplementing NSP-degrading enzymes, specifically endo-1,4-xylanase and β-glucanase, in reduced-energy corn–soybean meal diets (−100 kcal/kg AME) significantly influenced growth performance in a phase-dependent manner. During the grower phase (11–24 days), supplementation with both enzymes markedly improved FCR, ECR, and EPEF while reducing FCG. In the finisher phase (25–37 days), xylanase alone effectively improved FCR and reduced FCG, achieving results comparable to or superior to those of the PC diet. No adverse effects were observed on carcass yield, internal organ weights, breast meat pH, color, drip loss, or the incidence of WB and WS myopathies. These findings confirm that moderate dietary energy reduction, when combined with enzyme supplementation, maintains productivity and meat quality while reducing feed costs.

From a practical standpoint, this study provides a sustainable feeding strategy for commercial broiler production, particularly under tropical conditions where high-energy feed ingredients are expensive. The use of xylanase and β-glucanase during the grower phase, followed by xylanase alone during finishing, optimizes energy utilization from cereal NSP fractions, decreases dependency on dietary fat, and ensures high feed efficiency without compromising carcass traits or meat quality. The phase-specific supplementation approach also improves overall production economics and supports environmentally efficient poultry farming.

The study’s strengths lie in its comprehensive evaluation of enzyme efficacy across production phases, inclusion of both performance and meat quality parameters, and testing under commercial tropical rearing conditions. However, some limitations remain, including the short experimental duration, the lack of gut microbiome analysis, and the use of a single enzyme dosage and energy reduction level. Future research should focus on exploring different enzyme inclusion rates, combinations, and interactions with various cereal sources, as well as investigating microbial and metabolic responses that underpin improved nutrient utilization. In addition, studies examining gene expression related to energy metabolism, oxidative stress, and muscle physiology could further clarify the preventive role of enzyme supplementation in WB and WS myopathies.

The combined supplementation of xylanase and β-glucanase in reduced-energy broiler diets enhances feed efficiency, energy utilization, and economic returns without compromising carcass or meat quality. Phase-specific enzyme application represents an effective, science-based, and economically viable strategy for sustainable broiler production. By optimizing nutrient availability and moderating metabolic load, this feeding approach supports profitability, animal welfare, and integrity of meat quality, key goals for modern, efficient, and responsible poultry production systems.

## DATA AVAILABILITY

All the generated data are included in the manuscript.

## AUTHORS’ CONTRIBUTIONS

SV and PC: Conducted the study, performed data analysis, and drafted and revised the manuscript. PC, SN, and CR: Provided technical assistance during the experiments. CR, CK, and CB: Conception and design of the study and review of the manuscript. All authors have read and approved the final manuscript.

## References

[1] Choct M (2015). Feed non-starch polysaccharides for monogastric animals:Classification and function. Anim. Prod. Sci.

[2] Annison G, Hughes R.J, Choct M (1996). Effects of enzyme supplementation on the nutritive value of dehulled lupins. Br. Poult. Sci.

[3] Morgan N, Bhuiyan N.M, Hopcroft R (2022). Non-starch polysaccharide degradation in the gastrointestinal tract of broiler chickens fed commercial-type diets supplemented with either a single dose of xylanase, a double dose of xylanase, or a cocktail of non-starch polysaccharide-degrading enzymes. Poult. Sci.

[4] Habte-Tsion H.M, Kumar V, Rossi W (2018). Perspectives of non-starch polysaccharide enzymes in nutrition. In:Enzymes in Human and Animal Nutrition.

[5] Bedford M.R, Apajalahti J (2018). Exposure of a Broiler to a Xylanase for 35d Increases the Capacity of Cecal Microbiome to Ferment Soluble Xylan. Proceedings of Poultry Science Association..

[6] Munyaka P.M, Nandha N.K, Kiarie E, Nyachoti C.M, Khafipour E (2016). Impact of combined β-glucanase and xylanase enzymes on growth performance, nutrients utilization and gut microbiota in broiler chickens fed corn or wheat-based diets. Poult. Sci.

[7] Gilani S, Gracia M.I, Barnard L, Dersjant-Li Y, Millán C, Gibbs K (2021). Effects of a xylanase and beta-glucanase enzyme combination on growth performance of broilers fed maize-soybean meal-based diets. J. Appl. Anim. Nutr.

[8] Cerrate S, Ekmay R, England J.A, Coon C (2019). Predicting nutrient digestibility and energy value for broilers. Poult. Sci.

[9] Vieira S.L, Simões C.T, Kindlein L, Ferreira T.Z, Soster P, Stefanello C (2021). Progressive *in vivo* detection of wooden-breast in broilers as affected by dietary energy and protein. Poult. Sci.

[10] Thanatsang K.V, Malila Y, Arayamethakorn S, Srimarut Y, Tatiyaborworntham N, Uengwetwanit T, Panya A, Rungrassamee W, Visessanguan W (2020). Nutritional properties and oxidative indices of broiler breast meat affected by wooden-breast abnormality. Animals (Basel).

[11] Namted S, Bunchasak C (2022). Physical and chemical properties of meat in relation to wooden-breast myopathies in male broiler chickens raised in evaporative-cooling systems. Indian J. Anim. Res.

[12] Lake J.A, Abasht B (2020). Glucolipotoxicity:A proposed etiology for wooden-breast and related myopathies in commercial broiler chickens. Front. Physiol.

[13] Saleh A.A, Kirrella A.A, Abdo S.E, Mousa M.M, Badwi N.A, Ebeid T.A, Nada A.L, Mohamed M.A (2019). Effects of dietary xylanase and arabinofuranosidase combination on the growth performance, lipid peroxidation, blood constituents, and immune response of broilers fed low-energy diets. Animals (Basel).

[14] Aviagen-Ross-Broiler Management Handbook. https://en.aviagen.com/assets/tech/center/ross/broiler/ross/broilerhandbook2018-en.pdf..

[15] Oliveira R.F, Mello J, Ferrari F.B, Souza R.A, Pereira M.R, Cavalcanti E.N, Villegas-Cayllahua E.A, Fidelis H.A, Giampietro-Ganeco A, Fávero M.S, Souza P.A, Borba H (2021). Effect of aging on the quality of breast meat from broilers affected by wooden-breast myopathy. Animals (Basel).

[16] Kuttappan V.A, Brewer V.B, Mauromoustakos A, Mc-Kee S.R, Emmert J.L, Meullenet J.F, Owens C.M (2013). Estimation of factors associated with the occurrence of white-striping in broiler breast fillets. Poult. Sci.

[17] Duncan D.B (1955). Multiple range and multiple F-tests. Biometrics.

[18] Chen X, Zhang G.M, Wang W.W, Liu G.H, Cai H.Y, Purba A, Zheng A.J (2023). Compound non-starch polysaccharide enzymes improve growth performance, slaughter performance, immune function, and apparent utilization rate of nutrients in broiler chickens fed a low-metabolizable energy diet. Front. Vet. Sci.

[19] Demas G.E, Chefer V, Talan M.I, Nelson R.J (1997). Metabolic costs of mounting an antigen-stimulated immune response in adult and aged C57BL/6J mice. Am. J. Physiol.

[20] Luo D, Yang F, Yang X, Yao J, Shi B, Zhou Z (2009). Effects of xylanase on performance, blood parameters, intestinal morphology, microflora and digestive enzyme activities of broilers fed wheat-based diets. Asian J. Anim. Sci.

[21] Lamp A.E, Evans A.M, Moritz J.S (2015). The effects of pelleting and glucanase supplementation in hulled barleybased diets on feed manufacture, broiler performance, and digesta viscosity. J. Appl. Poult. Res.

[22] Samarasinghe K, Messikommer R, Wenk C (2000). Activity of supplemental enzymes and their effect on nutrient utilization and growth performance of growing chickens as affected by pelleting temperature. Arch. Tierernaehr.

[23] González-Ortiz G, Dos Santos T.T, Bedford M.R (2021). Evaluation of xylanase and a fermentable xylo-oligosaccharide on performance and ileal digestibility of broiler chickens fed energy and amino acid-deficient diets. Anim. Nutr.

[24] Alam M.J, Howlider M.A.R, Pramanik M.A.H, Haque M.A (2003). Effect of exogenous enzymes in diet on broiler performance. Int. J. Poult. Sci.

[25] Huang K.H, Li X, Ravindran V, Bryden W.L (2006). Comparison of apparent ileal amino acid digestibility of feed ingredients measured with broilers, layers, and roosters. Poult. Sci.

[26] EL-Sayed Z.S (2021). Effect of adding soy lecithin, xylanase and their combination to low-energy diet on carcass traits, meat quality, and some blood parameters of broiler chicks. Ann. Agric. Sci. Moshtohor.

[27] Singh A.K, Mishra B, Bedford M.R, Jha R (2021). Effects of supplemental xylanase and xylooligosaccharides on production performance and gut health variables of broiler chickens. J. Anim. Sci. Biotechnol.

[28] Ohotuowo K.O.O, Darlington I.E, Ogar O.P (2020). Enzyme supplementation of sorghum diets could influence the blood profile and histology of digestive organs in broiler chickens. EC Vet. Sci.

[29] Selim N.A, Abdel Magied A.H, Habib H.H, Waly H.A, Fadl A.A, Shalas S.M (2016). Effect of pectinase enzyme supplementation and low-energy corn–soybean meal diets on broiler performance and quality of carcass and meat. Egypt. Poult. Sci.

[30] Zakaria H.A.H, Jalal M.A.R, Ishmais M.A.A (2010). The influence of supplemental multi-enzyme feed additive on the performance, carcass characteristics and meat quality traits of broiler chickens. Int. J. Poult. Sci.

[31] Allouche L, Madani T, Hamouda Z.A, Boucherit M.R, Taleb H, Samah O, Rahmani K, Touabti A (2015). Effect of addition of exogenous enzymes in hypocaloric diet in broiler chicken on performance, biochemical parameters and meat characteristics. Biotechnol. Anim. Husb.

